# Origin of the Nervous System

**Published:** 1881-11

**Authors:** 


### ORIGIN OF THE NERVOUS SYSTEM.

The following statement of the results of the study of embryology in elucidating this obscure problem, was made by Mr. Balfour, at the last meeting of the British Association: 1. The nervous system of the higher Metazon has been developed in the course of a long series of generations by a gradual process of differentiation of parts of the epidermis. 2. Part of the central nervous system of many forms arose from a local collection of nerve cells in the epidermis, in the neighborhood of rudimentary organs of vision. 3. Ganglion cells have been evolved from simple epithelial cells of the epidermis. 4. The primitive nerves were outgrowths of the original ganglion cells, and the nerves of the higher forms are formed as outgrowths of the central nervous system.

